# Diabetes and hypertension increase the placental and transcellular permeation of the lipophilic drug diazepam in pregnant women

**DOI:** 10.1186/1471-2393-13-188

**Published:** 2013-10-17

**Authors:** Mladena Lalic-Popovic, Jovana Paunkovic, Zorica Grujic, Svetlana Golocorbin-Kon, Hani Al-Salami, Momir Mikov

**Affiliations:** 1 Department of Pharmacy, Faculty of Medicine, University of Novi Sad, Novi Sad, Serbia; 2 Health department in Novi Sad for women health protection, Novi Sad, Serbia; 3 Clinic of Gynecology and Obstetric, Clinical Centre of Vojvodina, Faculty of Medicine, University of Novi Sad, Novi Sad, Serbia; 4 Faculty of Pharmacy, University of Montenegro, Podgorica, Montenegro; 5 School of Pharmacy, Curtin Health Innovation Research Institute, Curtin University, Perth, WA, Australia; 6 Faculty of Health Science, Central Queensland University, Queensland, Australia; 7 Department of Pharmacology, Toxicology and Clinical Pharmacology, Faculty of Medicine, University Novi Sad, Novi Sad, Serbia

**Keywords:** Drug placental-permeation, Diazepam, Diabetes, Hypertension

## Abstract

**Background:**

Previous studies carried out in our laboratories have demonstrated impaired drug permeation in diabetic animals. In this study the permeation of diazepam (after a single dose of 5 mg/day, administered intramuscularly) will be investigated in diabetic and hypertensive pregnant women.

**Methods:**

A total 75 pregnant women were divided into three groups: group 1 (healthy control, n = 31), group 2 (diabetic, n = 14) and group 3 (hypertensive, n = 30). Two sets of diazepam plasma concentrations were collected and measured (after the administration of the same dose of diazepam), before, during and after delivery. The first set of blood samples was taken from the mother (maternal venous plasma). The second set of samples was taken from the fetus (fetal umbilical venous and arterial plasma). In order to assess the effect of diabetes and hypertension on diazepam placental-permeation, the ratios of fetal to maternal blood concentrations were determined. Differences were considered statistically significant if p ≤ 0.05.

**Results:**

The diabetes and hypertension groups have 2-fold increase in the fetal umbilical-venous concentrations, compared to the maternal venous concentrations. Feto: maternal plasma-concentrations ratios were higher in diabetes (2.01 ± 1.10) and hypertension (2.26 ± 1.23) groups compared with control (1.30 ± 0.48) while, there was no difference in ratios between the diabetes and hypertension groups. Umbilical-cord arterial: venous ratios (within each group) were similar among all groups (control: 0.97 ± 0.32; hypertension: 1.08 ± 0.60 and diabetes: 1.02 ± 0.77).

**Conclusions:**

On line with our previous findings which demonstrate disturbed transcellular trafficking of lipophilic drugs in diabetes, this study shows significant increase in diazepam placental-permeation in diabetic and hypertensive pregnant women suggesting poor transcellular control of drug permeation and flux, and bigger exposure of the fetus to drug-placental transport.

## Background

The development and progression of diabetes have been associated with disturbed drug absorption due to dysfunctional protein expression and functionality, impaired transcellular transport and intercellular trafficking as well as altered gut physiology [1].

Due to the widespread rise in early detection of high-risk pregnant women in need of cesareans, its use is rapidly increasing worldwide. High-risk pregnant women include those with uncontrolled diabetes, hypertension and pre-eclampsia [2-4]. Diazepam is used in the treatment of maternal eclampsia and as a premedication in cesarean section deliveries. It is highly lipophilic drug with linear pharmacokinetics [5]. Diazepam readily crosses the blood–brain barrier and the placenta by passive diffusion. It is also excreted into breast milk and recent studies show that diazepam reaches equilibrium in the feto-maternal systemic circulation 10-15 minutes after intravenous administration [6].

Passive diffusion rate depends on physicochemical properties of the drug; protein binding and state of placental barrier (i.e. blood flow). Some diseases such as diabetes and hypertension have been associated to impaired placental composition and functions. Moreover, pregnancy in diabetic and hypertensive women is directly linked to increased fetal morbidity and mortality [7-9]. These may be in a part related to changes in placental transport of maternal nutrients to the fetus and consequent abnormal placental development [10-13]. Placental dysfunction may result from a reduced placental blood flow and placental infarctions [14,15].

Despite the fact that diazepam has been used for many years in pregnancy, its direct placental permeation, in diabetic and hypertensive pregnant women, remains poorly understood. Thus the purpose of this study is to investigate the influences of diabetes and hypertension on the transplacental permeation of diazepam, after intramuscular injection to pregnant women.

## Methods

### Subjects

Pregnant women were recruited from the Gynecology and Obstetric clinic of Vojvodina (Serbia). Pregnant women scheduled for cesarean section, those who were diagnosed with gestational or arterial hypertension [16] as well as those who were diagnosed gestational diabetes [17] were included in this study. A total of 75 pregnant women were divided into three groups: healthy control group (n = 31), diabetes group (n = 14) and hypertension group (n = 30). Gestational or arterial hypertension was diagnosed according to The Good Clinical Practice National Guidelines on Diagnostic and Treatment of Arterial Hypertension [16]. Gestational diabetes mellitus was diagnosed according to The Good Clinical Practice National Guidelines on Diabetes Mellitus [17]. Three women in diabetes group were receiving insulin and the rest were on diabetes diet. The women in hypertension group were on methyldopa regular dosing. Prior to enrolment into the study, medical history was taken and a physical examination was performed. Only women with normal renal and liver function were included. Diazepam was applied by intramuscular injection (i.m.; 5 mg/day) into gluteal muscle of mothers. Maternal demographic data (i.e. age, height and weight) and neonatal birth weight, length and 1^st^ and 5^th^ minute Apgar scores were taken. The study protocol was approved by Ethic Committee of the Gynecology and Obstetistric clinic in Vojvodina (N°00-08/9) and informed consents were obtained from each participant before inclusion in the study. Before the cesarean section, a spinal anaesthesia with 0.5% bupivacaine hydrochloride or a general anaesthesia with propofol, succinylcholine and fentanyl were applied as a part of the clinic common procedures. Spinal anaestheisa was applied to 11 women in the control group, 2 women in the diabetes group and 3 women in the hypertension group. General anaesthesia was applied to 20 women in the control group, 27 women in the hypertension group and 12 women in the diabetes group.

### Sample collection

Maternal peripheral venous blood samples (2 mL) were collected from the arm using an indwelling cannula. Three samples were collected from all mothers. First sample was taken after application of diazepam and before cesarean section (time t_1_). Second sample of maternal blood was taken at the same time with clamping of the infant’s umbilical cord (time t_2_). Third sample was taken after cesarean section (time t_3_). Times t_1_, t_2_ and t_3_ are presented in Table 1. Neonatal umbilical cord venous and arterial samples were obtained from a section of umbilical cords, cross clamped at delivery. Blood samples were collected in a vacutainer containing EDTA.

**Table 1:** **Summary of a) feto: maternal diazepam plasma concentration ratios (C**_**v**_**/C**_**m**_**); b) umbilical cord arterial: venous diazepam plasma concentration ratios (C**_**a**_**/C**_**v**_**), and c) times of sample collection t**_**1**_**, t**_**2 **_**and t**_**3 **_**(min) (mean ± standard deviation (min-max))**

**Number of women [N = 75]**	**Control group [n = 31]**	**Hypertension group [n = 30]**	**Diabetes group [n = 14]**
**C**_**v**_**/C**_**m**_	1.30 ± 0.48 (0.59–2.27)	2.26 ± 1.23 (0.25–4.21)#	2.01 ± 1.10 (0.42–3.77) ##
**C**_**a**_**/C**_**v**_	0.97 ± 0.32 (0.1–1.60)	1.08 ± 0.60 (0.33–2.50)	1.02 ± 0.77 (0.17–2.97)
**t**_**1 **_**(min)**	25.9 ± 21.51 (5–118)	28.70 ± 18.18 (10–93)	20.31 ± 11.84 (7–42)
**t**_**2 **_**(min)**	60.23 ± 31.87 (18–144)	64.37 ± 22.69 (25–114)	45.38 ± 30.25 (18–138)
**t**_**3 **_**(min)**	132.39 ± 42.07 (67–230)	130.40 ± 39.90 (76–278)	122.38 ± 22.61 (92–172)

#control group vs. hypertension group, p ≤ 0.05.

##control group vs. diabetes group, p ≤ 0.05.

### Sample extraction

Plasma was transferred to plastic tubes after centrifugation and frozen at -20°C prior to analysis. Diazepam from plasma samples was extracted by liquid-liquid extraction. To 0.5 mL of plasma sample was added 0.5 mL of mixture of 5 M sodium hydroxide and 3 M sodium chloride. Mixture was vortexed for 30 seconds and then added 2.5 mL of chloroform for extraction and after vortexing 90 seconds sample was centrifuged 10 minutes at 3500 rpm (2100 rcf), 1.5 mL of the lower layer was transferred to glass tubes, evaporated at 60°C and before injection to HPLC system reconstituted with 100 μl of mobile phase. A 20 μl of sample was injected into HPLC system.

### Sample analysis

Diazepam concentrations in plasma were measured by modified HPLC method previously described [18]. HPLC system (*Dionex*) consisted of Agilent column (5 μm, 100 mm × 2.1 mm) with guard column (Agilent; 5 μm, 20 mm × 2.1 mm). Mobile phase was consisted of 50 mM potassium dihydrogen phosphate buffer (pH 3) and acetonitril in ration 65:35 (v/v), at flow rate 0.4 mL/min. Retention time for diazepam was 7.5 minutes. UV detection was set at 230 nm. Analyses were done at room temperature (25°C). The limit of detection (LOD) was 0.005 μg/mL and limit of quantification (LOQ) 0.01 μg/mL with recovery of 94.33 ± 2.12. The calibration curve was linear in the concentration range of 0.01-10 μg/mL.

### Statistical analysis

All data were measured as mean ± standard deviation (SD). Data were analyzed using one way analysis of variance (ANOVA) and the means were compared using Tukey`s test (P ≤ 0.05) via SPSS 17.0 (Systat Software Inc., San Jose, CA). Differences were considered significant if p ≤ 0.05. Areas under the curve (AUC) were calculated by trapezoidal method using WinNonLin (version 4.1; SCI software, Pharsight Corp., Gary NC, USA). Feto: maternal concentration ratios (c_v_/c_m_) were calculated by dividing diazepam concentrations in neonatal umbilical venous blood by concentrations in maternal venous blood. Also umbilical cord arterials to umbilical cord venous concentration ratios (c_a_/c_v_) were determined as a measure of diazepam uptake, distribution and/or metabolism in neonates.

## Results

### Demographic characteristics

There were total 75 women included in the study scheduled for cesarean section. All women went through normal pregnancy and gave birth to healthy single newborns. The age of included women was between 20 and 42 years. There were no statistically significant differences neither in height, weight nor body surface area (BSA; m^2^) of the women between three investigated groups (Table 2). Also, doses of diazepam normalized per body mass (mg/kg) and BSA (mg/m^2^) were not statistically different between groups (Table 2). However, systolic and diastolic pressures were statistically significantly higher in hypertension group compared to diabetes and control group as expected. All women in hypertension group were receiving methyldopa (250 mg) and in diabetes group 3 women were receiving insulin and the rest were on diabetes diet.

**Table 2:** Summary of demographic characteristics of mothers and dosage details (mean ± standard deviation (min-max)

**Number of women [N = 75]**	**Control group [n = 31]**	**Hypertension group [n = 30]**	**Diabetes group [n = 14]**
		**N = 30**	**N = 14**
Age [years]	31.03 ± 4.13 (23-40)	33.87 ± 5.72 (20-42)	32.14 ± 5.79 (24-41)
Height [m]	1.65 ± 0.078 (1.52–1.81)	1.66 ± 0.059 (1.51–1.80)	1.65 ± 0.065 (1.53–1.78)
Weight [kg]	79.97 ± 14.09 (60–136)	95.71 ± 22.69 (51–142)	87.00 ± 21.51 (60–127)
BSA [m^2^]*	1.87 ± 0.23 (1.09–2.59)	2.087 ± 0.28 (1.48–2.62)	1.97 ± 0.27 (1.61–2.45)
BMI [kg/m^2^]**	29.69 ± 6.65 (18.87–41.51)	32.66 ± 7.74 (19.2–43.2)	30.77 ± 7.47 (18.37–42.47)
Systolic pressure (mmHg)	111.3 ± 11.9 (90–130)	150.0 ± 14.3 (130–180) #	120.0 ± 16.4 (90–150)∆
Diastolic pressure (mmHg)	70.6 ± 8.5 (60–80)	96.7 ± 8.8 (80–120) #	73.6 ± 9.3 (60–90) ∆
Dose (mg/kg)	0.063 ± 0.0084 (0.04–0.08)	0.057 ± 0.014 (0.04–0.10)	0.061 ± 0.014 (0.04–0.08)
Dose (mg/m^2^)	2.66 ± 0.23 (1.93–3.12)	2.45 ± 0.35 (1.91–3.38)	2.58 ± 0.36 (2.04–3.11)

*Boyd ’ s equation BSA = 0.000327 * weight(g)(0.7285 - 0.0188 log10 weight(g)) * height(cm).

** BMI –body mass index; BMI = weight (kg)/height (m^2^).

#control group vs. hypertension group, p ≤ 0.05.

∆hypertension group vs. diabetes group, p ≤ 0.05.

All neonates were similar in length, weight and BSA (Table 3). Also Apgar scores for all the newborn babies, measured in 1^st^ and 5^th^ minute after birth, were mostly normal. In control group there were 2 neonates with 1^st^ minute Apgar score 6 and one neonate with 1^st^ minute Apgar score 5. Also, one newborn infant in the group of women with diabetes had 1^st^ minute Apgar score 6 and two neonates in group of women with hypertension had 1^st^ minute Apgar score 5. All neonates had 5^th^ minute Apgar score 7 or above. Statistically significant differences were observed in gestational age of children, where children in hypertension and diabetes group of women were delivered earlier. Transfer of diazepam starts from 6^th^ week of gestation and further its transfer is not dependant on gestational age thus the differences in gestational age between groups in this study could not influence diazepam transfer [19].

**Table 3:** Summary of demographic characteristics of neonates and Apgar scores (mean ± standard deviation (min-max))

**Number of women [N = 75]**	**Control group [n = 31]**	**Hypertension group [n = 30]**	**Diabetes group [n = 14]**
Length [cm]	49.84 ± 1.97 (45–53)	48.43 ± 3.03 (42–53)	49.43 ± 3.95 (41–54)
Weight [kg]	3.49 ± 0.51 (2.27–4.54)	3.12 ± 0.88 (1.73–4.65)	3.54 ± 0.75 (1.83–4.50)
BSA [m^2^]*	0.22 ± 0.0.02 (0.10–0.17)	0.21 ± 0.04 (0.13–0.14)	0.23 ± 0.03 (0.13–0.14)
Apgar score (1 min)	8.97 ± 1.36 (5–10)	7.9 ± 1.33 (5–10)	8.79 ± 1.26 (6–10)
Apgar score (5 min)	9.58 ± 0.83 (7–10)	8.83 ± 0.90 (7–10)	9.43 ± 0.82 (7–10)
Gestational age (weeks)	39.6 ± 0.8 (39–42)	37.9.1 ± 2.38 (32–41) #	38.3 ± 0.81 (37–39) ##
Neonatal gender (Male/Female)	14/17	22/8	8/6

#control group vs. hypertension group, p ≤ 0.05.

##control group vs. diabetes group, p ≤ 0.05.

*Boyd ’ s equation BSA = 0.000327 * weight (g) (0.7285 - 0.0188 log10 weight (g)) * height (cm).

### Pharmacokinetic analyses

**Figure 1:**
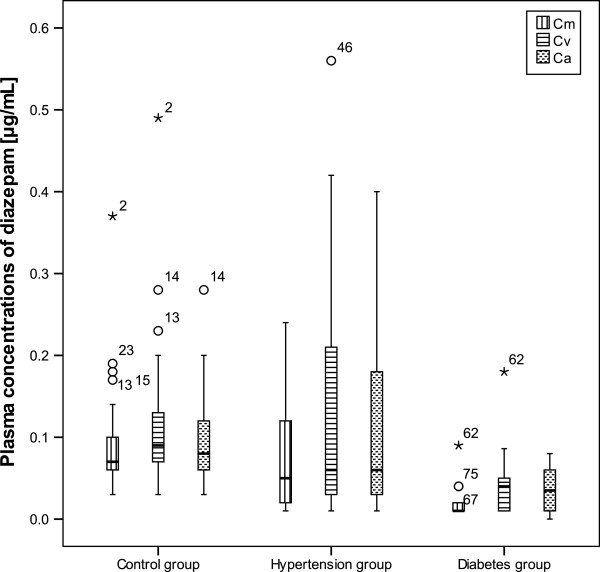
**Box plots of diazepam concentrations (μg/mL) in maternal venous plasma (C**_**m**_**) and neonatal venous umbilical cord (C**_**v**_**) and arterial umbilical cord (C**_**a**_**) plasma at delivery [black stars represent extreme cases and white circles are outlier values].**

The concentrations of diazepam (μg/mL) in the maternal plasma (c_m_) and neonatal plasma at delivery (venous umbilical cord plasma c_v_ and arteriala umbilical cord plasma c_a_) are shown in Figure 1. The corresponding feto: maternal plasma concentration ratios (c_v_/c_m_) and umbilical arterial: venous plasma ratios (c_a_/c_v_) are shown in Table 1. In the control group fetal diazepam concentrations were higher than maternal diazepam concentrations in 71% of cases (feto: maternal ratios > 1), while in hypertension group there were 80% of feto: maternal ratios higher than 1 and in diabetes group there were 78% feto: maternal ratios higher than 1. Maternal diazepam concentrations were statistically higher in control (0.09 ± 0.06 μg/mL) and hypertension group (0.07 ± 0.06 μg/mL) compared to group of women with diabetes (0.02 ± 0.01 μg/mL). Group of women with hypertension (0.12 ± 0.09 μg/mL) and control group (0.1 ± 0.05 μg/mL) had statistically higher arterial umbilical cord diazepam concentrations than group of women with diabetes (0.04 ± 0.03 μg/mL). There was statistically lower diazepam umbilical venous concentration in diabetes group (0.05 ± 0.04 μg/mL) compared to hypertension group (0.13 ± 0.12 μg/mL) and control group (0.11 ± 0.06 μg/mL). In control group 45% of arterial: venous cord ratios were over 1, while in diabetes and hypertension group 50% of arterial: venous cord ratios were over 1, where in hypertension group 13% and in diabetes group 7% of ratios were over 2, which was not detected in control.

Study was approved under condition that it did not disturb the regular clinical procedures, which resulted in sampling times variability (Table 1). However, in this study diazepam was applied by long needle (21 gauges, 40 mm) in the outer upper quadrant of the buttock by which it was avoided fat tissues thus it was expected to reach maximal concentrations after 0.30-1.30 hours [20-22].

**Figure 2:**
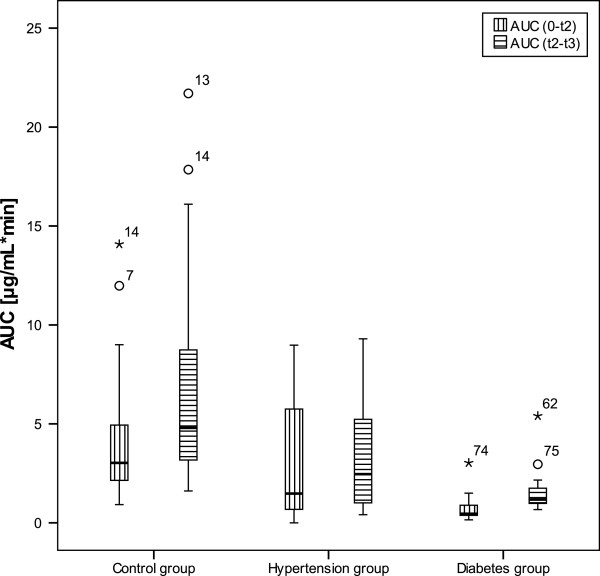
**Box plots of partial AUCs of diazepam in maternal venous plasma from the zero time to the time at delivery (AUC 0-t**_**2**_**) and from the time of delivery to the time of taken sample after cesarean section (AUC t**_**2**_**-t**_**3**_**) [black stars represent extreme cases and white circles are outlier values].**

In Figure 2 are presented partial area under the curve values (AUC; min*μg/mL) calculated from the zero time to the time at delivery (AUC 0-t_2_) and from the delivery time to the time of taken sample after cesarean section (AUC t_2_-t_3_). Values of AUC before delivery were taken as a measure of fetal exposure to diazepam. There were statistically higher AUC values before delivery in control and hypertension group compared to diabetes group. Values of AUC after delivery were statistically higher in control group compared to hypertension and diabetes group, but there were no significant differences between hypertension and diabetes group. Values of AUC after delivery were taken as a measure of diazepam elimination from the blood.

Diazepam concentrations in umbilical cord venous plasma, arterial cord plasma and maternal plasma were correlated with each other and weight, height, BSA, AUC and applied dose (Pearson’s coefficient of correlation were calculated; r^2^). Diazepam concentrations in umbilical cord venous plasma and concentrations in maternal venous plasma were positively correlated in all three groups (r^2^_=_ 0.75 control group; r^2^_=_ 0.80 diabetes group and r^2^_=_ 0.77 hypertension group, but in hypertension group three pairs were excluded from correlation). There were no correlations between dose, AUC, BSA, BMI, weight and maternal diazepam plasma concentrations at delivery (C_m_) or correlation between neonatal weight or BSA and umbilical cord plasma concentrations.

## Discussion

Main finding of this study is that feto: maternal ratios were higher in hypertension and diabetes group compared to control group. Maternal diazepam concentrations were statistically higher in control and hypertension group compared to group of women with diabetes. Umbilical cord arterial: venous plasma concentration ratios were similar in all groups and they were not statistically different.

The strength of this study is a prospective design which did not disturb regular clinical procedures. Limitation of this study is smaller number of pregnant women in diabetes group than in control and hypertension group.

In a majority of previous studies diazepam umbilical cord plasma concentrations exceeded maternal concentrations [23-25], although feto: maternal ratios below 1 have also been reported [26]. Diazepam is highly liposoluble and crosses placenta by passive diffusion via transcellular route [27,28] thus diazepam transfer depends on properties of the placenta and is in a function of multiple factors such as placental blood flow, protein binding, lipid solubility and ionization constant (pKa).

In our study there were 76% of feto: maternal ratios above and 24% of feto-maternal ratios below 1. There were also feto-maternal ratios above 2 (16% control group; 57% hypertension group; 36% diabetes group). This study demonstrates that feto: maternal ratios in diabetes and hypertension groups were significantly higher than in control. However, there was no difference between the ratios of the diabetes and hypertension groups. Since the administered doses of diazepam were similar in all groups, our findings show that there is a difference in diazepam transfer across placenta in the diabetes and hypertension groups.

There is no drug-drug interactions observed or reported [29-32] between diazepam and other administered drugs/anaesthetics, which indicates that diazepam absorption studies were not compromised.

Since no known interaction on pharmacokinetic level are expected between diazepam and insulin it was not considered that insulin treatment in 3 women in diabetes group had effect on concentration data.

Interestingly, in one study, a single oral dose of 5 mg diazepam taken before dental treatment did not influence blood glucose level in nondiabetic and non-insulin dependent diabetic subjects [33]. But diazepam may alter insulin secretion and insulin sensitivity after a single administration in healthy volunteers [34], and diazepam-induced hyperglycemia, might be related to changes in serum chromium levels [35]. However in this study the increase in glycemia in diabetic women would be the result of the stress due to operation rather than diazepam treatment.

Binding of diazepam to human serum albumin can be influenced by hypertension and diabetes due to changes in hemodynamic properties or by drugs used for their treatment. Since diazepam is highly bound to proteins (96-99%), and protein bound fraction of drug do not cross placenta [36], its metabolism rate and transplacental passage depends on free drug fraction. Diazepam is more protein bound in fetal circulation than in maternal circulation and it has one binding site on human serum albumin [37]. Since there is no known interactions at the level of protein binding between methyldopa or insulin nor drugs used in anaesthesia, observed higher feto: maternal ratios in diabetes and hypertension group could be consequence of the increased level of some endogenous substance in diabetes and hypertension, like free fatty acid which displace diazepam from proteins [38,39].

In the study by Ridd et al. [39] were found differences in protein binding of diazepam in maternal and diazepam plasma but free diazepam fractions were similar on both sides of placenta. Unbound drug concentrations in plasma are responsible for the obtained pharmacological effects, thus it would be expected that partial displacement of diazepam from plasma proteins would result in the increase in the magnitude of its pharmacological effects [40-43]. In our study there were no side effects in newborns (i.e. Apgar scores had normal values) or in mothers but this does not imply that there were no increase in diazepam free fraction. Since total clearance of diazepam is directly proportional to free diazepam fraction [44-46] and increased free diazepam fraction would lead to higher elimination and disappearance of diazepam from blood. This could be proved through AUC values after delivery which were statistically higher in control group compared to hypertension and diabetes group that implies that elimination of diazepam from central compartment is higher in hypertension and diabetes group and that is likely that there were more unbound diazepam in the blood in these groups.

Also other mechanisms such as metabolism and active transport could lead to differences in feto: maternal unit. Diazepam is metabolized by CYP enzymes some of which are present in placenta like CYP3A4 [47]. Presence of CYP 3A4 in human placenta is more than controversial, based on original and review articles [48], although some expression may be present at a very low, not functionally significant level. The most direct study on the absence of CYP3A-catalyzed metabolism was conducted on carbamazepine [49]. Previous studies showed that diazepam is not substrate for P-glycoprotein [50] but recent study suggested that diazepam is a modifier of P-glycoprotein because it behaved as an activator of the P-glycoprotein ATP-ase activity [51]. The potential of diazepam to modify P-glycoportein could affect placental transfer of other drugs which are substrates of P-glycoprotein. Still observed higher transplacental transition of diazepam in diabetes and hypertension group in our study may be due to lower activity of some other efflux proteins.

Ionic composition of cellular and extracellular space is altered in hypertension and diabetes due to altered work of some ion exchange pumps like Na^+^/H^+^ and Ca^2+/^H^+^[52,53]. This could have influence on degree of diazepam ionization since if ionic strength of solution is lower dissociation balance shift to neutral molecules. Namely, diazepam is weak base (pKa 3.3), mainly unionized in blood (pH 7.4) when unionized molecules leave plasma balance is shifted to new ionization. Since fetal plasma is slightly more acidic, 0.1 lower than maternal plasma pH, the un-ionized free drug crossing the placenta becomes ionized and is 'trapped’ in the more acidic fetal circulation [54,55]. In this study only in control group in all cases umbilical cord arterial: venous diazepam plasma concentration ratios were close to one, but in hypertension and diabetes group half of ratios were over one which means that higher maintenance of diazepam occurred within infants of mothers with diabetes or hypertension. As it was single dose use of diazepam and Apgar scores were not statistically different between groups there were no observed differences in pharmacological effect on neonates between groups. Some studies showed that maternal diazepam medication affects the beat-to-beat variability in the newborns [56] but duration of the effect is more profound in the chronic and infusion diazepam groups. However, it is known that diazepam free fraction increases shortly after birth which explains adverse effects observed clinically in some diazepam exposed neonates [57]. Thus it could be concluded that hypertensive and diabetic group neonates were in higher risk of serious diazepam side effects.

## Conclusions

As expected, our findings show significant and direct effect of diabetes and hypertension development on diazepam absorption kinetics and transcellular trafficking across the placenta. On the other hand, the findings do not show a significant difference between diabetes and hypertension in diazepam placental-permeation. This raises further questions on the suitability of lipophilic drugs in pregnancy and future short and long term safety of the new born babies. Thus further studies are necessary to determine whether other lipophilic drugs also show such difference in placental-permeation.

## Competing interests

The authors declared that they have no competing interests.

## Authors’ contributions

MLP carried out HPLC measurements and participated in analysis and interpretation of data, the design and writing of the manuscript. JP collected samples and participated in the design and writing of the manuscript. ZG supervised implementation of the study and participated in the design and writing of the manuscript. SGK and HAS participated in the design and writing of the manuscript. MM supervised the experiments, implementation of the study and participated in analysis and interpretation of data, the design and writing of the manuscript. All authors have seen and approved the final version.

## Pre-publication history

The pre-publication history for this paper can be accessed here:

http://www.biomedcentral.com/1471-2393/13/188/prepub
